# Maize Silage Associated With Hay From Xerophytic Plants in Lamb Feeding: Intake, Digestibility, and Economic Feasibility

**DOI:** 10.1111/asj.70181

**Published:** 2026-05-12

**Authors:** Rosilda Alves Magalhães Menezes, Mario Adriano Ávila Queiroz, Fleming Sena Campos, Juliana Caroline Santos Santana, Péricles Brito Batista, Rafael Torres de Souza Rodrigues, Higor Fábio Carvalho Bezerra, Severino Gonzaga Neto, Timóteo Silva dos Santos Nunes, Willames Luiz do Nascimento, Lucas Lopes de Macedo, João Virgínio Emerenciano Neto

**Affiliations:** ^1^ Federal Institute of Education, Science and Technology of the Sertão Pernambucano Petrolina Pernambuco Brazil; ^2^ Federal University of Vale São Francisco Petrolina Pernambuco Brazil; ^3^ State University of Southwest Bahia ‐ Vitória da Conquista Bahia Brazil; ^4^ Federal University of Rio Grande do Norte Macaíba Rio Grande do Norte Brazil; ^5^ Federal University of Paraíba Areia Paraíba Brazil

**Keywords:** carcass yield, conservation, *Gliricidia sepium*, *Leucaena leucocephala*, *Manihot*

## Abstract

The aim of this study was to evaluate the effect of the partial inclusion of 25% hay from xerophytic plants in maize silage in feedlot lamb in the semiarid region. Non‐defined breed male lambs were used in a completely randomized design, with five experimental units per treatment. The treatments consisted of four diets: maize silage (SM), maize silage with gliricidia hay (SMG), maize silage with pornunça hay (SMP), and maize silage with leucaena hay (SML). The SM diet showed higher in vivo dry matter digestibility (70.35%), whereas SM and SMG recorded the highest NDF digestibility values (60.31% and 57.10%). Average daily gain was higher in the SM and SMG diets (180 g day^−1^), resulting in greater slaughter weights (31.39 and 29.74 kg). Carcass yield was not affected by the inclusion of xerophytic plant hays. Gliricidia maintained carcass performance similar to SM, whereas SMP and SML resulted in lower carcass weights and commercial cuts. Meat color improved with the inclusion of legumes (SMG and SML). The economic analysis indicated that SM was the most profitable diet, although all diets showed positive profit. Gliricidia is a viable alternative for semiarid production systems, contributing to feed diversification and reducing dependence on maize silage.

**Abbreviations**
a*: red–green color component
ADF: acid detergent fiber
ADG: average daily gain
b*: yellow–blue color component
BFT: backfat thickness
CCD: cold carcass dressing
CCY: cold carcass yield
CP: crude protein
CPD: crude protein digestibility
CPI: crude protein intake
DM: dry matter
DMD: dry matter digestibility
DMI: dry matter intake
EE: ether extract
EOC: effective operating cost
FBW: final body weight
FCR: feed conversion ratio
GM: gross margin
GR: gross revenue
HCD: hot carcass dressing
HCY: hot carcass yield
L*: meat lightness
LMA: *Longissimus* muscle area
NDF: neutral detergent fiber
NDFD: neutral detergent fiber digestibility
NDFI: neutral detergent fiber intake
NFC: nonfibrous carbohydrates
NM: net margin
OC: opportunity cost
SM: maize silage
SMG: maize silage with gliricidia hay
SML: maize silage with leucaena hay
SMP: maize silage with pornunça hay
TC: total cost
TCD: true carcass dressing
TDN: total digestible nutrients
WHC: water holding capacity

## 1 Introduction

Feeding is the costliest component in animal production, regardless of species or production system, and may account for up to 70% of total costs (Makkar 2013). Therefore, nutritional strategies that reduce expenses without compromising animal performance are essential for the sustainability of livestock systems. Among these strategies, the use of high‐quality conserved diets stands out, as they ensure a continuous feed supply throughout the year and reduce dependence on concentrate ingredients, which usually have high costs.

Conservation through silage and haymaking helps mitigate the effects of forage seasonality, ensuring a continuous feed supply and supporting animal maintenance throughout the year (Neres et al. 2021). This strategy becomes even more relevant under the edaphoclimatic conditions of semiarid regions, which directly affect forage availability and farm profitability. Thus, forage conservation practices are especially important, particularly through proper management of Caatinga vegetation and the use of forages adapted to this environment (Silva and Cézar 2013). In feedlot systems, where diets often include grains and maize silage, dependence on this input can increase feeding costs. The partial inclusion of conserved ingredients from drought‐adapted plants represents a promising alternative to reduce reliance on maize silage, provided that animal productivity is maintained.

Maize silage is one of the main ingredients used in feedlot diets, but its production and purchase can substantially increase system costs. In this context, the partial inclusion of conserved ingredients from drought‐adapted forage species emerges as an alternative to reduce dependence on maize silage and make feeding systems more cost‐effective. However, any ingredient used must be able to maintain animal productivity, because reductions in performance compromise feedlot efficiency.

Several forage species adapted to semiarid conditions have been highlighted as viable alternatives for ruminant feeding. Among these xerophytic plants, gliricidia ( *Gliricidia sepium* ), leucaena ( *Leucaena leucocephala* ), and pornunça (Manihot spp.) show relevant nutritional potential and can be used either as hay or silage. The inclusion of these plants in the diet can improve production quality by supporting ruminal fermentation, animal performance, and carcass traits (Ferraretto et al. 2018). In addition, they represent strategic feed reserves for the Brazilian semiarid region, helping to maintain feed supply and production stability during periods of forage deficit (Fluck et al. 2018; Voltolini et al. 2019).

In this context, the objective of this study was to evaluate the effects of the partial inclusion of hay from drought‐tolerant forage species in maize silage on intake, digestibility, performance, carcass characteristics, and economic viability of feedlot lambs raised in a semiarid environment.

## 2 Materials and Methods

### 2.1 Animal Welfare Statement

This study was approved by the Animal Ethics Committee (CEUA) of the Federal University of Vale do São Francisco (UNIVASF), under protocol number 0007/230518 (2018).

### 2.2 Experimental Site

The experiment was conducted at the Laboratory of Animal Requirements and Metabolism (LEMA), located at the Campus of Agricultural Sciences of the Federal University of Vale do São Francisco, Petrolina, PE, Brazil (9°19′19.29″ S, 40°33′40.99″ W; 393 m above sea level). According to the Köppen classification, the climate is hot semiarid with a rainy season (BSh), with a mean annual precipitation of 700 mm, irregularly distributed. The dry period lasts approximately 9 months, with rainfall concentrated between February and April. In the region, annual temperatures range between 24°C and 28°C, with marked seasonal variation.

### 2.3 Experimental Design, Animals, and Diets

Treatments consisted of four diets: maize silage (SM), maize silage plus gliricidia hay (SMG), maize silage plus pornunça hay (SMP), and maize silage plus leucaena hay (SML), with five replicates per treatment. Twenty intact male lambs with no defined breed pattern were used, with an initial body weight of 18 ± 2 kg and an age of 5 ± 0.5 months. The feedlot period lasted 70 days, preceded by a 15‐day adaptation period. Animals were housed in individual pens (1.0 m × 2.0 m × 1.0 m) equipped with feeders and drinkers.

Animals were fed diets formulated with a forage‐to‐concentrate ratio of 70:30, in which maize silage (70%) was partially replaced by 25% hay from drought‐tolerant forage species (45% maize silage and 25% hay in the SMG, SMP, and SML diets) (Table 1). Diets were balanced to support an average daily gain of 150 g/day, considering an estimated dry matter intake (DMI) of 1.05 kg/day (NRC – National Research Council 2007). Feed was offered twice daily, at 08:00 and 15:00 h, at an amount equivalent to 5% of body weight, with daily adjustments based on observed intake and the maintenance of approximately 10% refusals. Additional adjustments were made after animal weighing, correcting the amount offered in g of dry matter (DM) per kg of body weight.

**TABLE 1:** Ingredients and chemical composition of the experimental diets.

Ingredient (%)	Diet			
	SM	SMG	SMP	SML
Maize silage	70.00	45.00	45.00	45.00
Gliricidia hay	—	25.00	—	—
Leucaena hay	—	—	—	25.00
Pornunça hay	—	—	25.00	—
Urea	0.70	0.70	0.70	0.70
Maize grain	16.35	20.20	21.70	18.60
Mineral mix	2.00	2.00	2.00	2.00
Soybean meal	9.15	3.05	4.85	8.45
Wheat bran	1.80	4.05	0.75	0.25
Chemical composition, % of dry matter				
Dry matter	47.61	64.39	64.37	63.69
Ash	6.00	7.16	7.01	7.07
Crude protein	14.55	14.00	14.85	14.20
Ether extract	3.49	3.23	3.51	3.46
Neutral detergent fiber	37.65	38.10	36.18	36.80
Acid detergent fiber	17.03	18.27	18.76	18.98
Nonfibrous carbohydrates	39.58	38.78	39.72	39.74
Total digestible nutrients	68.09	67.90	68.70	68.44

Abbreviations: SM, maize silage; SMG, maize silage with gliricidia hay; SML, maize silage with leucaena hay; SMP, maize silage with pornunça hay.

### 2.4 Diet Preparation, Sampling, Intake, and Digestibility

For diet preparation, the silage was produced using whole maize ( *Zea mays* ) chopped into particle sizes of 10–25 mm. The material was packed using a LABOREMUS SBL35n bagging machine into plastic bags (75 × 105 cm, 200 μm thickness) with a capacity of 35 kg and fermented for 30 days.

Hays were produced from *Gliricidia sepium* , *Manihot* sp., and *Leucaena leucocephala* . The plants were identified and separated into leaves and stems using pruning shears and a handsaw. Only the leaves of the leguminous plants were used for hay production, based on their higher crude protein (CP) content and faster dehydration rate compared with the stems. The material was chopped using a stationary forage chopper (JF 50 Maxxium) and sun‐dried on plastic tarpaulins, separated by treatment, and turned every 2 h until reaching approximately 10% moisture over a 2‐day period, under ambient temperatures ranging from 23°C to 32°C, according to Bayão et al. (2016). The hay was then stored until diet formulation.

Samples of the offered diets were collected and sent to the Animal Nutrition and Bromatology Laboratory for determination of chemical composition (Table 1). The samples were individually ground in a knife mill (Willey Mill, Marconi MA‐580), pre‐dried in a forced‐air oven at 55°C for 72 h, and analyzed according to AOAC (2000) for DM (method 967.03), CP (method 981.10), and ether extract (EE; method 920.29). Neutral detergent fiber (NDF) and acid detergent fiber (ADF) were determined according to Mertens et al. (2002) and Licitra et al. (1996).

Total digestible nutrients (TDN) were estimated using the equation: TDN = 83.79 − (0.4171 × NDF), as described by Cappelle et al. (2001). Nonfibrous carbohydrates (NFC) were calculated as follows: NFC = 100 − ash − EE − NDF − (CP − CPu + U), where ash, EE, NDF, CP from urea (CPu), urea (U), and CP were used.

### 2.5 Feed Intake and Digestibility

DMI (kg day^−1^) was calculated daily as the difference between the amount of feed offered and orts from the previous day. Based on these values and the chemical composition of the diets (Table 1), crude protein intake (CPI, kg day^−1^) and neutral detergent fiber intake (NDFI, kg day^−1^) were also determined.

In vivo digestibility was evaluated during the last 7 days of the experiment. The animals were adapted to the use of fecal collection bags for 3 days before the start of collections. During the final 4 days, feces were collected twice daily, homogenized, and weighed. After each collection, 10% of the daily fecal output was sampled, identified, and stored at −15°C. At the end of the period, the samples were thawed, pre‐dried in a forced‐air oven at 55°C for 72 h, ground, and sieved through a 1‐mm screen for laboratory analyses.

The digestibility coefficients of DM, CP, and NDF were calculated according to Schneider and Flatt (1975), using the equation:

$$\text{Digestibility}\;\left(\%\right)=\frac{\text{Nutrient intake}-\text{Nutrient excreted}}{\text{Nutrient intake}}\times 100$$

### 2.6 Animal Performance and Carcass Evaluation

Animals were weighed at the beginning of the experiment, every 7 days, and at the end of the experimental period after a 14‐h solid feed withdrawal. Average daily gain (ADG, g day^−1^) was calculated as the difference between initial and final body weight (FBW) divided by the experimental period, and feed conversion ratio (FCR) was obtained as the ratio between DMI and ADG. FBW (kg) corresponded to the value recorded at the last weighing prior to slaughter.

After 70 days of experimental feeding, the animals were subjected to a 16‐h solid fasting period, in accordance with the current regulations of the Brazilian Regulation for Industrial and Sanitary Inspection of Products of Animal Origin (Brasil 2017). Animals were stunned using a captive bolt pistol and slaughtered following animal welfare recommendations. Carcasses were skinned, eviscerated, trimmed, and weighed to obtain hot carcass weight. Subsequently, carcasses were suspended by the Achilles tendon and chilled for 24 h at 4°C, when cold carcass weight was determined. Slaughter weight is the live body weight of the animals at the time of slaughter, measured after a 16‐h solid feed withdrawal. Empty body weight was calculated as the live body weight minus the weight of the gastrointestinal tract contents and internal organs.

Hot carcass yield percentage (HCY, %) was calculated as the ratio between hot carcass weight and slaughter weight, cold carcass yield percentage (CCY, %) as the ratio between cold carcass weight and slaughter weight, and true carcass yield percentage as the ratio between hot carcass weight and empty body weight, all multiplied by 100.

The left half‐carcass was weighed and sectioned between the 12th and 13th ribs, and subcutaneous fat thickness (SFT, mm) was measured using a digital caliper. The *Longissimus* muscle area (LMA, cm^2^) was obtained by tracing the contour of the *Longissimus dorsi* muscle on millimeter paper and subsequently processing the image using AutoCAD, according to standard methodology. Thereafter, commercial cuts of the left half‐carcass were obtained, including shoulder, neck, ribs, leg, flank, and loin, following anatomical limits described in the literature. Each cut was individually weighed to determine its respective yield. All portions were identified, packaged in plastic bags, and stored at temperatures between −12°C and −18°C until further analyses.

Meat analyses were performed after thawing the portions corresponding to the *Longissimus dorsi* muscle. Color was evaluated after 24 h of thawing using a portable colorimeter (Konica Minolta CR‐400) operating in the CIELAB system. Readings were taken 30 min after exposing the muscle surface to air, allowing myoglobin oxygenation. Meat lightness (L*) was recorded as a brightness value ranging from 0% (black) to 100% (white), while the a* component indicated color variation from green (−a) to red (+a), and the b* component represented variation from blue (−b) to yellow (+b), according to CIE Lab (1986) specifications.

After color measurements, CP, moisture, and ash content of the meat were determined according to official AOAC (2000) methods, with moisture determined by method 967.03, CP by method 981.10, and ash by method 942.05. Water‐holding capacity (WHC) was evaluated according to the methodology of Hamm (1961), based on the force required to expel water retained in the muscle matrix under applied pressure. Muscle pH was measured directly in the *Longissimus dorsi* using a portable penetration pH meter, following the procedures described by Grandis et al. (2016).

### 2.7 Economic Analysis of Diet and Carcass Production

The economic analysis of diet production and carcass yields was conducted according to the recommendations of SENAR (2014). Equal unit values were considered across diets for costs related to animal purchase, family labor, transport, slaughter and hide, health management, income tax rate, labor remuneration (social charges + hourly/daily wage of total labor), depreciation, health expenses, opportunity cost of invested capital, inputs, gross carcass, and electricity per day. Other economic components varied among diets according to specific feeding costs, mainly those associated with the production and acquisition of hays and maize silage.

Based on these values, economic indicators were determined. The operating cost was obtained by summing direct expenses with inputs, family labor, and depreciation. Total cost included the operating cost plus the opportunity cost of capital. Gross revenue was calculated by multiplying the processed carcass weight by the market price per kilogram. Gross margin was determined as the difference between gross revenue and operating cost, whereas net margin corresponded to gross revenue minus total cost. Profit was obtained as the difference between gross revenue and the sum of the total costs considered in the production system. Average cutting cost represents the mean cost related to carcass processing and fabrication into commercial cuts. Feed cost was calculated separately for each diet based on the quantity of ingredients consumed and their respective market prices during the experimental period. This item included the costs associated with maize silage production or acquisition and the production or procurement of xerophytic hays. All values reflected market prices practiced during the experimental period. To allow international comparison, all financial values were converted from Brazilian reais (R$) to US dollars (US$), using an exchange rate of US$ 1 = R$ 5.38.

### 2.8 Statistical Analysis

The results were subjected to analysis of variance (ANOVA), considering a completely randomized design, with four experimental diets (SM, SMG, SMP, and SML) and five replicates per treatment. Analyses were performed using the Statistical Analysis System software (SAS Institute Inc 2004), following the model:

$$\mathrm{Yij}=\mu +\mathrm{ti}+\mathrm{Ɛij};\mathrm{Ɛij}\;\mathrm{IID}∼N\;\left(0;\sigma 2\right),$$

where *Yij* represents the observed value for the *i*th treatment in the *j*th replicate, *μ* is the overall mean, *ti* corresponds to the fixed effect of treatment, and *εij* is the random error. When a treatment effect was detected (*p* < 0.05), Tukey's test at the 5% probability level was applied for multiple comparisons among means.

## 3 Results and Discussion

The diets affected DMI and CPI. The inclusion of gliricidia hay increased DMI, whereas pornunça hay increased CPI (Table 2). NDFI was not affected by hay inclusion, remaining constant at a mean of 0.40 kg day^−1^, indicating that the fibrous fraction intake was not altered by diet composition.

**TABLE 2:** Intake and digestibility of lamb fed maize silage associated with hays from drought‐tolerant plants.

Variables		Diets			SEM	*p*
	SM	SMG	SMP	SML		
Intake (kg day^−1^)						
Dry matter intake	1.16b	1.54a	1.06b	1.19b	0.22	< 0.01
Crude protein intake	0.14b	0.13b	0.15a	0.14b	0.03	< 0.01
Neutral detergent fiber intake	0.37	0.43	0.37	0.44	0.05	0.55
Digestibility (%)						
In vivo dry matter digestibility	70.35a	66.34b	63.47b	62.11b	5.86	< 0.01
In vivo CP digestibility	75.45	71.00	78.79	77.12	1.95	0.3510
In vivo NDF digestibility	60.31a	57.10a	54.87b	52.08b	2.35	< 0.01

*Note:* Means followed by different lowercase letters within rows differ by Tukey's test at 5% probability.

Abbreviations: CP, crude protein; NDF, neutral detergent fiber; SEM, standard error of the mean; SM, maize silage; SMG, maize silage with Gliricidia hay; SML, maize silage with Leucaena hay; SMP, maize silage with Pornunça hay.

Higher CPI in the SMP diet (0.15 kg day^−1^) was associated with the higher CP content of this formulation (Table 1). All diets exceeded the predicted intake of 1.05 kg day^−1^, showing that legume hays did not limit feed intake. This response indicates adequate acceptability of gliricidia, pornunça, and leucaena hays in lamb diets based on maize silage.

Dry matter digestibility (DMD) was higher in the SM diet (70.35%) compared with the other diets (mean of 63.97%) (Table 2). Crude protein digestibility (CPD) did not differ among diets (mean of 75.59%). Neutral detergent fiber digestibility (NDFD) was higher for SM (60.31%) and SMG (57.10%) than for SMP (54.87%) and SML (52.08%), which showed similar values.

This reduction in DMD and NDFD may be associated with the higher proportion of ADF relative to NDF in the hay‐based diets (Table 1). The unfavorable ADF:NDF ratio (51.9% for SMP and 51.6% for SML) indicates that the fibrous fraction of these diets is mainly composed of cellulose and lignin, which are components of the ADF fraction with low ruminal degradability, while exhibiting a lower contribution of hemicellulose, a more readily degradable fraction (Foster et al. 2011; Santana et al. 2022).

ADF is the cell wall component most closely related to limitations in fiber digestibility, and its increase reduces the ruminal disappearance of this fraction (Pereira et al. 2017). Thus, even with moderate NDF levels, the higher ADF content limits cell wall digestion, explaining the lower digestibility observed in the SMP and SML diets.

It is important to highlight that this moderate reduction in digestibility (approximately 3–4 percentage points) did not compromise productive performance, because the SMG diet maintained an average daily gain equivalent to the SM diet (180 g day^−1^; Table 3). In addition, the observed ADG was within the target established during diet formulation, which recommended a gain of 150 g day^−1^, according to NRC—National Research Council (2007).

**TABLE 3:** Performance of lamb fed maize silage associated with hays from drought‐adapted plants.

Variables	Diets				SEM	*p*
	SM	SMG	SMP	SML		
Average daily gain (g day^−1^)	180a	180a	150b	150b	0.03	< 0.01
Feed conversion ratio	6.54b	8.43a	6.93b	7.99a	1.49	< 0.01
Slaughter weight (kg)	31.39a	29.74a	28.71b	28.45b	0.26	< 0.01
Half carcass weight (kg)	7.28a	7.10a	6.55a	5.58b	0.32	0.05

*Note:* Means followed by different lowercase letters within rows differ by Tukey's test at 5% probability.

Abbreviations: CP, crude protein; NDF, neutral detergent fiber; SEM, standard error of the mean; SM, maize silage; SMG, maize silage with Gliricidia hay; SML, maize silage with Leucaena hay; SMP, maize silage with Pornunça hay.

The highest ADG values were observed in animals fed the SM and SMG diets (180 g day^−1^), whereas SMP and SML showed lower values (150 g day^−1^) (Table 3). Feed conversion was superior in the SM and SMP diets, with values of 6.54 and 6.93, respectively. Although the SMG diet resulted in higher DMI, this increase did not translate into improved productive efficiency, because the ratio between intake and weight gain was less favorable, and the higher efficiency observed in the SMP diet was not reflected in a greater slaughter weight.

Efficiency measures do not directly affect carcass yield or quality; however, they are useful to identify animals capable of producing with lower feed intake, thereby improving overall system efficiency. Nevertheless, some more efficient animals may display low weight gain; therefore, it is essential to consider intake, efficiency, and performance simultaneously (Gurgeira et al. 2022).

The highest slaughter weights were observed in animals fed the SM (31.39 kg) and SMG (29.74 kg) diets. Animals fed the SMP and SML diets were slaughtered at weights of 28.71 kg and 28.45 kg, respectively. The higher ADG observed in the SM and SMG diets resulted in greater slaughter weights. The SM and SMG diets showed greater fiber digestion efficiency, which may have contributed to the higher ADG and, consequently, greater slaughter weight.

The increase in NDF digestibility improves the supply of energy and metabolizable protein of microbial origin, which favors greater nitrogen retention and, consequently, higher weight gain in animals (Detmann et al. 2024). The highest half‐carcass weights were recorded for SM (7.28 kg), SMG (7.10 kg), and SMP (6.55 kg), whereas the SML diet resulted in the lowest value (5.58 kg), approximately 1.39 kg below the mean observed for the other diets (6.97 kg). The SMP diet promoted higher CPI; however, this was not reflected in greater CPD, nor in higher average daily gain and slaughter weight, as also reported by Tang et al. (2025), who found no effect on animal performance when comparing diets with high and low concentrations of rumen undegradable protein.

The inclusion of gliricidia hay in the diet maintains productive indices similar to those observed in exclusive maize silage, whereas pornunça hay and, especially, leucaena hay reduce average daily gain, which is reflected in lower slaughter weight of the animals.

Carcass yield of lamb was not affected by the inclusion of gliricidia, pornunça, or leucaena hays, remaining similar to the diet composed exclusively of maize silage (SM) (Table 4). The observed means were 49.08% for hot carcass yield (HCY), 47.52% for cold carcass yield (CCY), and 59.86% for true carcass yield (TCY). These values were within the range considered ideal for lamb, which is from 40% to 52% (Schreurs and Kenyon 2017). Despite the stability in carcass yields, the diets influenced the weight of commercial cuts. Animals fed the SML diet showed lower values for shoulder (1.02 kg), neck (0.54 kg), ribs (1.46 kg), leg (1.91 kg), and flank (0.28 kg).

**TABLE 4:** Carcass yield and physicochemical characteristics of meat from lamb fed diets containing maize silage and hays of drought‐adapted plants.

Variables	Diets				SEM	*p*
	SM	SMG	SMP	SML		
Carcass yield (%)						
HCY (%)	50.60	49.40	47.93	48.41	0.70	0.59
CCY (%)	48.60	47.90	46.72	46.84	0.12	0.55
TCY (%)	61.10	59.40	59.73	59.21	0.49	0.65
Commercial cuts (kg)						
Shoulder (kg)	1.25a	1.21a	1.20a	1.02b	0.05	0.01
Neck (kg)	0.80a	0.65ab	0.58b	0.54b	0.04	0.01
Ribs (kg)	1.96a	1.93a	1.68b	1.46b	0.09	0.03
Leg (kg)	2.32a	2.30a	2.15a	1.91b	0.08	0.03
Flank (kg)	0.37a	0.41a	0.36a	0.28b	0.02	0.01
Loin (kg)	0.59	0.62	0.64	0.42	0.04	0.06
SFT (mm)	2.16	2.49	2.28	2.14	0.08	0.52
LMA (cm^2^)	14.00a	14.30a	13.32a	11.51b	0.30	0.01
Meat after thawing						
L*	36.30b	40.50a	38.63ab	41.18a	0.64	0.01
a*	13.10	15.20	14.47	14.74	0.34	0.21
b*	2.35b	3.50ab	3.35ab	3.99a	0.18	0.02
Crude protein (%)	20.10	20.0	19.80	20.27	0.19	0.35
Moisture (%)	73.90	75.00	75.23	74.46	0.75	0.25
Ash (%)	1.08	1.09	1.19	1.09	0.03	0.53
WHC (%)	64.90	63.40	65.80	64.51	0.58	0.50
pH	5.73	5.59	5.81	5.64	0.04	0.58

*Note:* Means followed by different lowercase letters within rows differ by Tukey's test at the 5% probability level.

Abbreviations: a*, green–red component; b*, blue–yellow component; CCY, cold carcass yield; HCY, hot carcass yield; L*, lightness; LMA, *Longissimus* muscle area; SEM, standard error of the mean; SFT, subcutaneous fat thickness; SM, maize silage; SMG, maize silage with Gliricidia hay; SML, maize silage with Leucaena hay; SMP, maize silage with Pornunça hay; TCY, true carcass yield; WHC, water‐holding capacity.

Diets SM, SMG, and SMP maintained similar weights for shoulder, leg, flank and LMA, indicating that the inclusion of gliricidia and pornunça preserves the development of the main cuts with higher commercial value. Exclusive maize silage and the diet containing gliricidia presented the highest rib weights (1.96 and 1.93 kg, respectively), demonstrating the close productive response between SM and SMG.

The LMA is an important indicator of the muscularity of the *Longissimus dorsi*. Higher values were observed in animals fed SM (14.00 cm^2^), SMG (14.30 cm^2^), and SMP (13.32 cm^2^), whereas SML resulted in the lowest LMA (11.51 cm^2^). The inclusion of leucaena hay reduced carcass muscularity, while gliricidia and pornunça maintained performance similar to SM.

SFT did not differ among diets, with an average of 2.27 mm. As all diets presented the same forage‐to‐concentrate ratio (70:30), this result was expected. In lamb, diets with a higher proportion of forage tend not to alter fat thickness (Grandis et al. 2016). Therefore, the inclusion of legume hays did not affect carcass fat finishing.

The diets influenced meat color after thawing. Lightness (L*) was higher in SMG (40.50) and SML (41.18), indicating lighter meat. Higher L* values reflect greater lightness, which is considered desirable, as very dark meat is often associated with oxidative processes, darkening or defects such as DFD (dark, firm, dry) (Khliji et al. 2010). Animals fed SMP showed intermediate values (38.63), while SM showed the lowest lightness (36.30), resulting in darker meat, but still within the acceptable range, because L* values above 34 are considered visually acceptable to consumers (Khliji et al. 2010).

The increase in lightness in diets containing gliricidia and leucaena hay may be related to greater color stability of the meat, possibly due to the presence of natural antioxidant compounds in these legumes, such as tannins and flavonoids, which help to reduce myoglobin oxidation and delay the formation of metmyoglobin, preserving color and preventing darkening after thawing (Priolo et al. 2002; Brito et al. 2016).

The a* parameter (redness) did not vary among diets, with a mean of 14.38, within the typical range for young lamb meat (12 to 18) (Kadim et al. 2004). Maintaining consistent redness is important for the visual perception of meat freshness and indicates that, despite differences in lightness, the quality of red color was not compromised by the different diets. The a* values observed in this study fall within the range considered acceptable for consumer acceptance. According to Khliji et al. (2010), meat with a* values below 9.5 tends to present a dark and unattractive color, which was not observed in the evaluated diets.

The b* parameter, which expresses the tendency towards yellow color, was higher in the SML diet (3.99), followed by SMG (3.50) and SMP (3.35), and was lower in SM (2.35). This pattern suggests that legume hays, especially leucaena, intensify the yellow tone of lamb meat. This effect may influence consumer preference depending on cultural context. Valued attributes vary among countries: Consumers in Northern Europe tend to prefer meat from pasture‐fed lambs with a stronger flavor, whereas in Southern Europe (Greece, Italy and Spain), preference is for meat from milk‐fed or concentrate‐fed lambs with a milder flavor (Sañudo et al. 2007).

In the Brazilian context, where acceptance of lamb meat from forage‐based systems predominates, the moderate increase in the b* parameter, as observed in SML (3.99), is compatible with the national consumption profile and may even be interpreted as an indicator of production based on natural feeds. Lambs maintained exclusively on pasture tend to show lighter and more yellow fat than those finished in confinement (Priolo et al. 2002), due to the greater accumulation of carotenoids derived from forage (Prache et al. 2022).

Chemical parameters of the meat, including CP, moisture, ash, water‐holding capacity and pH, were not affected by the diets. The observed means were CP 20.04%, moisture 74.66%, ash 1.11%, water‐holding capacity 64.65%, and pH 5.69. These values remained within the ranges commonly reported for lamb muscle, characterized by CP contents between 18% and 22% and moisture between 72% and 76%. The observed pH remained within the recommended range for lamb meat, generally between 5.5 and 5.8 (Teixeira et al. 2005). The pH values were below the upper limit considered safe for microbiological stability, which is 5.8 (Egan and Shay 1988), indicating an adequate post‐mortem acidification process. This pattern, with final pH below 5.8, is compatible with efficient post‐mortem glycolysis and suggests that the animals were not subjected to marked pre‐slaughter stress, a condition associated with the attainment of a lower and more desirable pH (Gardner et al. 1999; Brito et al. 2016). Therefore, the partial replacement of maize silage with gliricidia, pornunça, or leucaena hays did not compromise the nutritional quality of the meat.

Operational and total costs were identical among diets (US$ 397.98 and US$ 402.27), as they included only fixed items common to all treatments, such as animal purchase, family labor, depreciation, animal health, and the opportunity cost of capital, according to the methodology proposed by SENAR (2014) (Table 5). Thus, the observed economic differences resulted exclusively from productive performance, especially carcass weight and the amount of saleable meat.

**TABLE 5:** Economic analysis of meat lamb production fed maize silage and hays from drought‐adapted plants.

Item (US$)	Diets			
	SM	SMG	SMP	SML
Operating cost	397.98	397.98	397.98	397.98
Total cost	402.27	402.27	402.27	402.27
Feed cost	149.77	156.24	102.86	116.01
Average cutting cost	49.51	48.64	46.90	38.60
Processed carcass (US$/kg)	6.79	6.83	6.91	6.79
Carcass revenue	495.39	486.15	456.23	382.42
Gross revenue	990.89	973.01	1033.34	724.92
Gross margin	660.14	361.00	352.84	207.40
Net margin	593.11	293.29	284.99	139.66
Profit	587.63	287.71	278.88	134.08

Abbreviations: SM, maize silage; SMG, maize silage with Gliricidia hay; SML, maize silage with Leucaena hay; SMP, maize silage with Pornunça hay.

However, the partial replacement of maize silage with xerophytic plant hays represents a strategic option for semiarid regions. Although immediate accounting costs were similar among diets, the use of gliricidia, pornunça, and leucaena may reduce dependence on external feed inputs and improve feed security, even when short‐term economic indicators remain balanced.

Thus, under the conditions evaluated, profitability was driven mainly by animal performance rather than by reductions in feeding costs. The xerophytic plants remain strategically relevant in semiarid systems, as they increase flexibility in feed resources and reduce reliance on maize under conditions of price volatility or forage scarcity.

The SM diet presented the best economic outcome, with the highest profit (US$ 587.63), the highest gross margin (US$ 660.14), and the highest net margin (US$ 593.11). Among the hay‐based diets, SMG was the most efficient, with a profit of US$ 287.71, followed by SMP (US$ 278.88), while SML presented the lowest return (US$ 134.08). These results directly reflect the higher carcass weights and greater commercial yield associated with SM.

Reductions in the weight of individual cuts, mainly observed in SMP and SML, although discrete, resulted in lower final weights of the half‐carcasses and reduced total revenue. The SML diet presented the lowest return precisely because it combined the lowest slaughter weight with the lowest carcass production. In contrast, SM generated the highest revenue per carcass (US$ 495.39), due to its superior productive performance.

Gross revenue varied according to the value of the processed carcass. However, even though the SMP treatment achieved a higher price per kilogram (US$ 6.91), this did not offset the superior productive performance of SM, which maintained the highest economic margins.

This pattern confirms that SMP and SML diets, in addition to producing lower carcass weights, also extended the time required to reach slaughter weight, thereby increasing the opportunity cost. According to Voltolini et al. (2019), lower cost diets do not necessarily ensure higher profitability when animal performance is compromised, which reinforces the pattern observed in this study.

The results demonstrate that, under the evaluated conditions, SM used alone promotes the highest economic return. The inclusion of gliricidia, pornunça, or leucaena hays is technically feasible; however, profitability is reduced when animal performance declines. Therefore, the evaluation of alternative feeding strategies in meat lamb systems should simultaneously consider feed costs and their effects on animal performance, especially in semiarid regions. This response was expected, as maize silage–based diets tend to be more profitable due to their high digestibility and favorable feed conversion (McDonald et al. 1991). SM achieved the best productive performance, carcass quality, and economic return. Among hay‐based diets, gliricidia (SMG) was the most efficient alternative, maintaining good animal performance with carcasses comparable to those obtained with SM. Gliricidia, pornunça, and leucaena hays showed good acceptability, indicating that all three species can be used in ruminant feeding, although with lower productive efficiency than SM.

The inclusion of hays is a viable strategy for ruminant production, but it requires careful evaluation of its effects on carcass yield when the objective is to maximize financial return. Nevertheless, the use of xerophytic plants represents a valuable alternative in semiarid regions, where maize prices fluctuate due to climatic and geopolitical factors. Alternative diets that maintain performance close to SM, as observed with gliricidia, reduce dependence on maize and offer greater economic stability. Therefore, the inclusion of gliricidia, pornunça, and leucaena hays can be adopted as a strategy for partial replacement of maize silage in lamb feedlot systems, particularly in environments subject to water deficit.

The inclusion of 25% hay in maize silage affected the productive and economic performance of feedlot lamb. The inclusion of gliricidia hay maintained zootechnical performance similar to that of the diet based exclusively on maize silage, whereas pornunça reduced productivity and leucaena resulted in the greatest reduction in animal performance. The inclusion of gliricidia, pornunça, and leucaena hays did not alter carcass yield and maintained carcass quality within the standards expected for feedlot‐finished lamb.

## Conflicts of Interest

The authors declare no conflicts of interest.

## Data Availability

Data available on request from the authors.
